# Progress in Psychiatry

**Published:** 1902-01-18

**Authors:** 


### Progress in Psychiatry.

(*Continued from page 255.*)

**Prodromal Symptoms.**—The prodromal stages of melancholia vary in length in different subjects. In some the depression of spirits never passes the limits of sanity as in the class of cases above described by Lange. In others the onset is gradual, commencing with anorexia, constipation, and gastro-intestinal disorder. A persistent tendency to worry in regard to health, business, or family affairs, and a disregard for work and for personal tidiness may have existed for some time. A combination of these two sets of circumstances may gradually usher in melancholia, without exciting suspicion in the patient's friends, until an attempt at suicide or other dangerous or destructive impulse suddenly calls attention to the condition of mental alienation present. In others again the onset may be of rapid occurrence following upon some serious shock or stress operating on a predisposed subject, as after seduction and desertion in a neurotic girl. A woman at the period of the menopause is liable to become melancholic and to develop delusions more readily than at other times. Thus in Ziehen's statistics of 238 cases of melancholia, 19·3 per cent. occurred between the fortieth and fiftieth years of life, and of these 12·6 per cent. were women and 6·7 per cent. were men. In the two decades from 40 to 60 years were included 37 per cent. of the total number of cases, 24 per cent. being men and 13 per cent. women. In the two decades from 15 to 35 years were included 30·7 per cent. of the cases, 11 per cent. being males and 19·7 per cent. being females. As the disease tends to become established nutritive defects of the body become more evident. The skin becomes of a dry muddy tint and feels unusually dry and harsh to the touch. Perspiration is absent or very slight indeed. A slight degree of jaundice may exist. The mouth and lips are dry, the tongue furred and flabby, the bowels constipated, and the breath tends to become offensive. Gastric digestion is much below par. The urine is small in quantity with traces of albumen and loaded with phosphates, appetite is lost, and loss of weight occurs. The pulse is slow, the arteries contracted and the radial tension increased. Sleep is profoundly disturbed, a condition of distressing insomnia being combined with frightful dreams and nightmare. Headache, feelings of tenderness about the scalp and nuchal spine, and fleeting neuralgias in various parts of the body are prominent symptoms. In women the menstrual process is arrested or the normal flow is decreased. The mental symptoms include slowness in thought and flow of ideas, vague anxiety, and fear of everything around, disinclination for work or initiative of any kind, an undue tendency to mental fatigue and confusion as the result of mental exertion, and a persistent brooding on and absorption in the feelings of misery, fear, and suffering. The period of mental lucidity does not last long, for delusions tend to develop with great facility when the disease is as yet in an early stage. These are slight and fleeting at first but tend to become persistent after a while.

**Delusions.**—Well-marked delusions, essentially of a painful nature, are only present in the more severe forms of melancholia. They are not infrequently started by the gastro-intestinal disturbances or the somatic pains and paræsthesiæ present. The concentration of the mind upon the *ego*, and the inactivity of the kinæsthetic life, tend to foster moods favourable to delusions. Among the commoner forms of delusion are those of being poisoned, or being damned or lost, of being actually in hell, of having committed the unpardonable sin of having committed murder or the death of others, of being ruined, disgraced, and deserted, or of being a disgrace and burden to those near and dear, and generally of personal unworthiness and merited misery and suffering. Acute melancholia may have various aspects, and in some patients several of these may be variously combined and blended. Hence we have the conditions of melancholia agitans, m. attonita with a tendency to stupor, delusional melancholia of the hypochondriacal form, m. with impulses to suicide or less frequently to homicide, m. with crises of terror shown in flight, movements of shrinking and self-defence, and finally the form described by Cotard under the title of *délire de negation* in which the development of delusions regarding the *ego* is carried to its ultimate stage, viz., the belief in the annihilation and non-existence of the affected part or whole of the *ego*. The acute manifestations of melancholia sometimes blend with symptoms of mania so as to make it difficult to distinguish between the two. Hence Kraepelin[6] has proposed for such attacks the title "*manische-depressive Irresein.*" The mental pain and misery experienced by the patient may be manifested in tears and groans, weeping and wringing of the hands, sorrowful utterances and ejaculations such as, "O God!" "I am lost and in hell," and the like, or in pathetic attitudes expressive of misery, fear, suffering or abject lowliness and unworthiness.

[6] Psychiatrie, 1900.

